# Thymic adenocarcinoma presenting as an incidental mediastinal mass

**DOI:** 10.1186/s13019-022-02000-8

**Published:** 2022-10-06

**Authors:** Anne E. O’Shea, Alexander P. Nissen, Donnell K. Bowen, Taylor L. Barnett, Joshua D. Gustafson

**Affiliations:** 1 Department of Surgery, San Antonio Military Medical Center, Fort Sam Houston, TX USA; 2 Division of Cardiothoracic Surgery, San Antonio Military Medical Center, 3551 Roger Brooke Drive, Fort Sam Houston, TX 78234 USA; 3 Division of Hematology and Oncology, San Antonio Military Medical Center, Fort Sam Houston, TX USA

**Keywords:** Thymus, Thymic adenocarcinoma, Thymectomy, Acute myeloid leukemia

## Abstract

**Background:**

Primary thymic adenocarcinoma represents an exceptionally rare malignancy, for which the cornerstone of therapy is margin-negative resection, with radiation and systemic therapy reserved for invasive and advanced disease. Thymic adenocarcinoma has not been previously reported in the setting of a concomitant malignancy, as reported herein.

**Case presentation:**

We present a case of a 55-year-old previously healthy male diagnosed with acute myeloid leukemia, also found to have a mediastinal mass. Evaluation of the mediastinal mass with tumor markers, biopsies, and next-generation sequencing proved non-diagnostic, while he was simultaneously treated with induction chemotherapy to prevent leukemia-related blast crisis. After completing and recovering from induction chemotherapy, he underwent successful thymectomy during a chemotherapy holiday, with a margin-negative resection of thymic adenocarcinoma. He has subsequently recovered and undergone successful allogeneic hematopoietic stem cell transplant.

**Conclusions:**

We present a case of synchronous adult acute myeloid leukemia and primary thymic adenocarcinoma requiring a tailored approach for management of simultaneous malignancies.

## Background

The incidence of primary thymic adenocarcinoma is extremely rare and has never been described as a synchronous diagnosis with a second primary malignancy [1]. We report an unusual case of primary thymic adenocarcinoma identified incidentally during a workup for newly diagnosed adult acute myeloid leukemia (AML).

## Case presentation

**Fig. 1:**
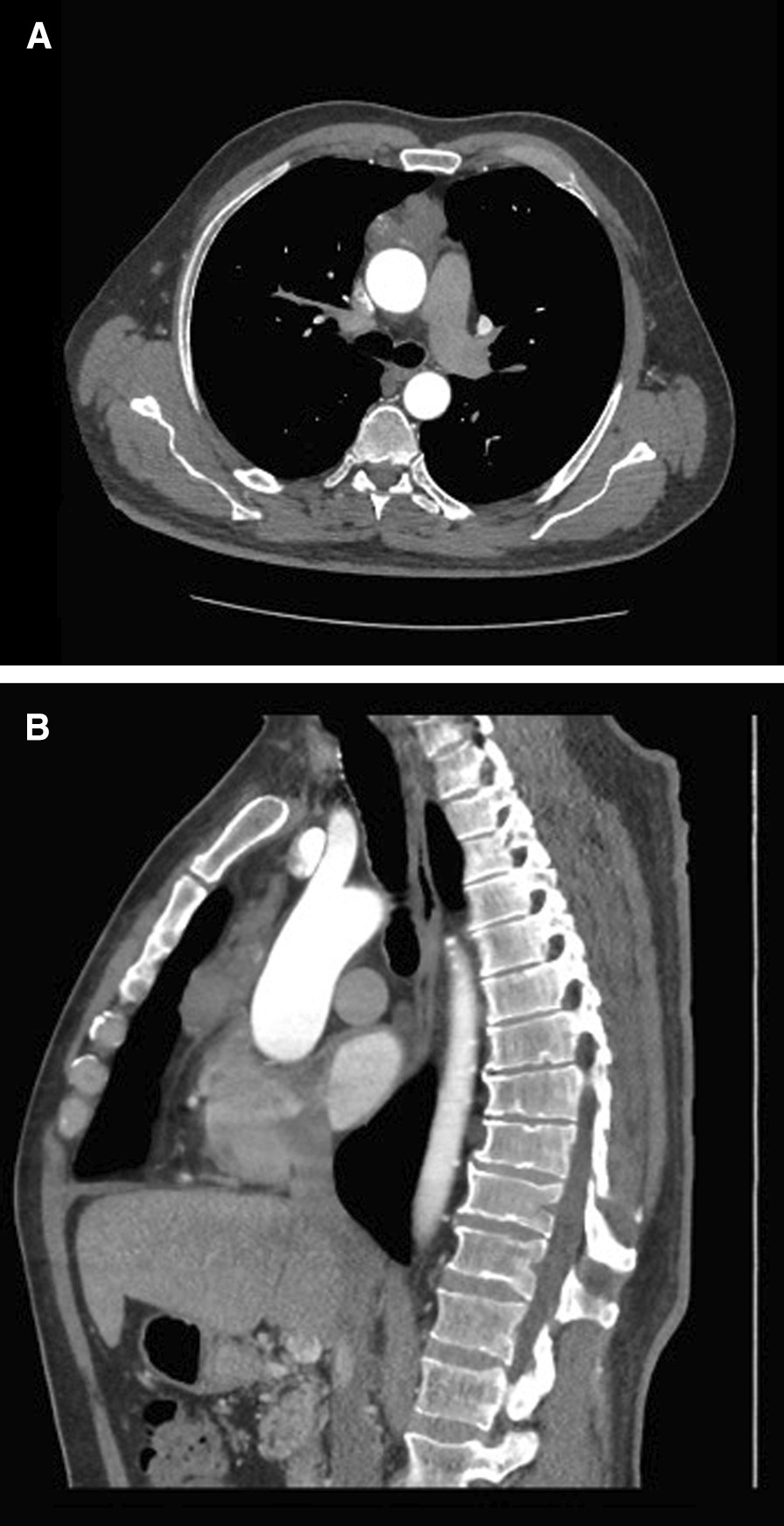
Axial (**A**) and Sagittal (**B**) representative CT images of the anterior mediastinal mass with abutment of the aorta and pericardium

A 55-year-old man presented to the Emergency Department with chest discomfort, fever, and fatigue. He had profound leukocytosis (> 58,000/µl) and elevated peripheral blast count (85%) concerning for acute leukemia. He had no other past medical history or family history of cancer. Following bone marrow aspiration to confirm the diagnosis of high-risk AML (> 95% blasts), chest computed tomography (CT) was obtained, demonstrating a 5.5 cm heterogeneous anterior mediastinal mass abutting the aorta (Fig. 1A, B). The patient underwent a CT-guided biopsy of the anterior mediastinal mass with pathology demonstrating carcinoma of unknown origin. The mass stained positive for pan-cytokeratin (Lu-5), cytokeratin-20 (CK20), and intestinal differentiation (CDX2), and negative for thyroid transcription factor 1 (TTF1)/Napsin, prostatic origin (NKX3.1), octamer binding transcription factor 4 (OCT4), glypican, and synaptophysin. All serologic tumor markers to include carcinoembryonic antigen (CEA), carbohydrate antigen (CA) 19-9, alpha fetoprotein (AFP), beta-human chorionic gonadotropin (HCG), prostate-specific antigen (PSA), and lactate dehydrogenase (LDH) were normal. Positron emission tomography-CT (PET-CT) demonstrated heterogenous fluorodeoxyglucose (FDG)-avidity within the mediastinal mass (max SUV 3.8), diffusely increased marrow signal appropriate in the setting of AML, and no other focus of increased FDG avidity. A second attempt at mediastinal CT-guided biopsy was similarly unsuccessful at determining the origin, in spite of next generation sequencing and tumor origin analysis.

After multi-disciplinary discussion, the patient began induction chemotherapy for treatment of his high-risk FMS-like tyrosine kinase (FLT) 3 AML with idarubicin, cytarabine, and midostaurin. This course was complicated by neutropenic fever, clostridium difficile colitis, appendicitis, and culture-negative tricuspid valve endocarditis. After recovery, a repeat bone marrow biopsy and aspirate demonstrated a complete molecular remission. The patient had appropriate hematologic recovery and maintained good performance status. Repeat CT scan of the chest demonstrated no significant change in the mediastinal mass.

**Fig. 2:**
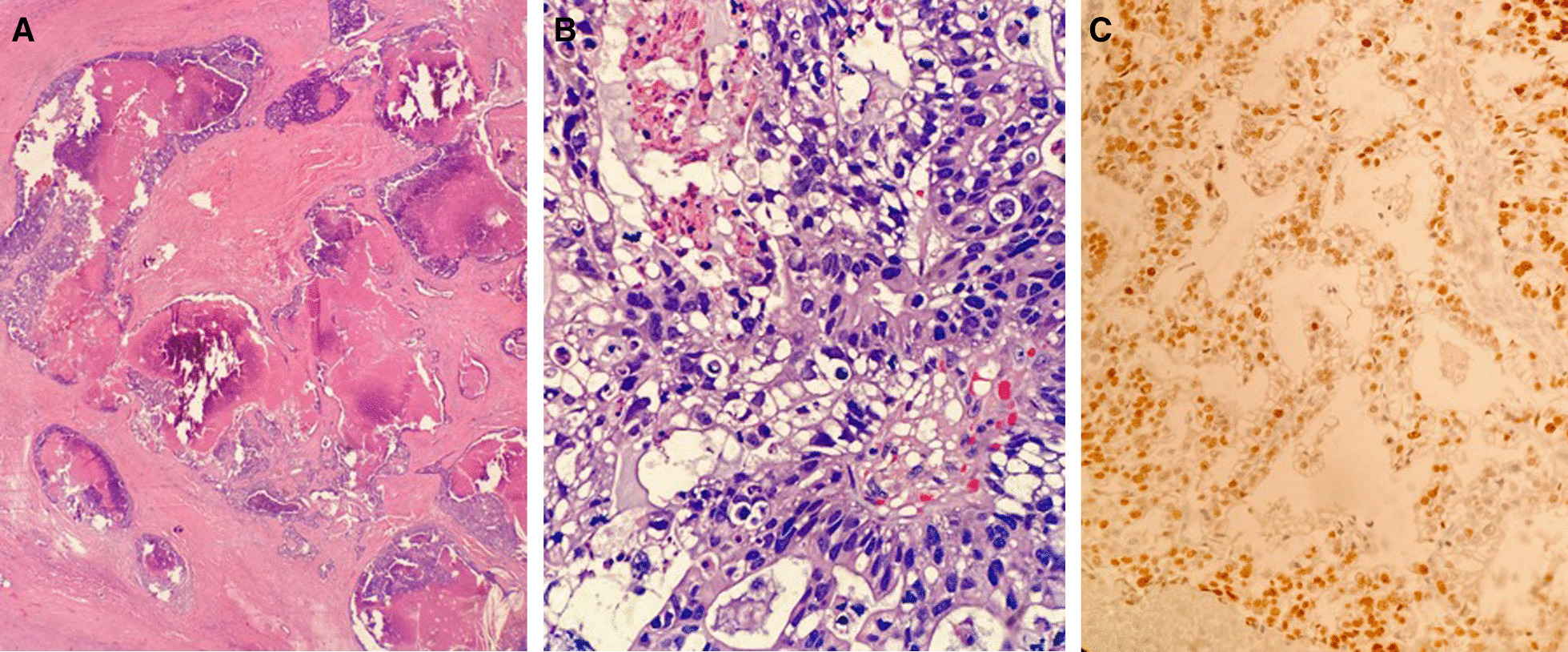
**A** Low power view showing multiple epithelial lined cysts separated by fibrous stroma with pools of tumor necrosis. **B** High power view showing pleiomorphic epithelial cells with prominent nucleoli, mitotic figures, and apoptotic cells. (2C) CDX2 stain, a specific marker of intestinal differentiation, showing focal and varying positivity in the lesional nuclei

He subsequently underwent open thymectomy via sternotomy for both diagnostic and therapeutic purposes after a 4-week chemotherapy holiday. Standard thymectomy was performed, including removal of both cervical horns, all tissue between the phrenic nerves, and inferiorly to the diaphragm, to include a portion of the right pleura to which the mass was adherent. The deep aspect of our dissection demonstrated a free plane between the mediastinal mass and the aorta, as well as the pericardium. Final pathology demonstrated margin-negative resection of a primary thymic adenocarcinoma, 6.5 cm in greatest dimension, with tumor staining consistent with the preoperative core needle biopsies (Fig. 2A–C). Additional commercial genetic testing of 91 candidate genes was negative for identifiable pathogenic mutations, variants of unknown significance, or gross deletions or duplications. There was no evidence of local invasion, with 0 of 10 lymph nodes involved, yielding pathologic Stage 1 (pT1N0M0), Masaoka-Koga Stage 1, thymic adenocarcinoma. This was confirmed on expert pathological send-out review.

The patient has recovered well and is now more than 3 months from surgery. Adjuvant radiation was considered but ultimately deferred to avoid further AML-related systemic therapy delays. He restarted consolidation chemotherapy four weeks postoperatively given his high-risk AML and has now undergone successful allogeneic hematopoietic stem cell transplant from a matched unrelated donor.

## Discussion and conclusions

Thymic epithelial tumors represent a collection of rare malignancies, occurring at a frequency of less than one in 100,000 persons annually [1, 2]. Under the umbrella of thymic carcinomas, squamous, adenosquamous/mucoepidermoid, and undifferentiated represent the most common subtypes. Primary thymic adenocarcinomas are particularly rare, with fewer than 30 reported in the literature. None were previously diagnosed in the setting of a second synchronous malignancy [3]. The majority of patients are asymptomatic at the time of diagnosis, while the remainder typically note nonspecific chest discomfort and/or dyspnea as their primary complaint.

The cornerstone of management for localized thymic carcinomas remains R0 resection followed by close surveillance. Adjuvant radiation therapy and/or chemotherapy are reserved for more advanced or recurrent disease [4]. While chemotherapy, primarily with platinum or anthracycline-based regimens, remains an option for advanced thymic carcinomas, additional complicating factors in this case include the concomitant administration of cytotoxic therapy for AML. Use of idarubicin in the treatment of AML may limit subsequent candidacy for additional anthracyclines in the event of thymic carcinoma recurrence due to cumulative dose-related cardiotoxicity [2, 5]. Thymic adenocarcinomas also typically do not respond completely to cytotoxic chemotherapy.

Collectively, these factors created a situation of nuanced prioritization of both diagnosis and rapid initiation of curative-intent treatment for AML to prevent complications including blast crisis, infection, and coagulopathic bleeding, while simultaneously ruling out metastatic adenocarcinoma, teratoma, or germ cell tumor, prior to proceeding with thymectomy during an appropriately timed chemotherapy holiday. Ultimately, this tailored approach allowed for good short-term outcome while balancing the exceptionally rare instance of primary thymic adenocarcinoma and adult AML.

## Data Availability

N/A.

## Abbreviations

AML: Acute myeloid leukemia
CT: Computed tomography
PET: Positron emission tomography
FDG: Fluorodeoxyglucose
